# Late-Life Changes in Hypothalamic-Pituitary-Testosterone Axis, Health, and Survival in Men With Longevity

**DOI:** 10.1093/geroni/igaf122.3617

**Published:** 2025-12-31

**Authors:** Noah Gold, Mary Biggs, Michelle Odden, Diana He, Anne Newman, Alvin Matsumoto, Sofiya Milman, Sandra Aleksic

**Affiliations:** Albert Einstein College of Medicine, Bronx, New York, United States; University of Washington, Seattle, Washington, United States; Stanford School of Medicine, Palo Alto, California, United States; University of Pennsylvania, Philadelphia, Pennsylvania, United States; University of Pittsburgh, Pittsburgh, Pennsylvania, United States; University of Washington School of Medicine, Seattle, Washington, United States; Albert Einstein College of Medicine, Bronx, New York, United States; Albert Einstein College of Medicine, Bronx, New York, United States

## Abstract

While lower testosterone levels in middle-aged men have been associated with poorer metabolic health and mortality, similar associations were not demonstrated in our previous work in nonagenarians and centenarians. As more men reach longevity, it is important to characterize late-life trajectory and etiology of testosterone decline, and their relationship with health and survival. Among 134 men in the Cardiovascular Health Study who had reached 85+ years of age (mean 88±3 years) by the 2005-6 visit, we measured total testosterone (TT; mass spectrometry), luteinizing hormone (LH) and sex hormone-binding globulin (SHBG) (immunoassay) in serum from 2005-6 and another visit 9-13 years earlier. Free testosterone (FT) was calculated. Mean age at death was 93±4 years. Between two visits, TT did not change (463±187 vs. 437±219 ng/dL, p = 0.17). However, FT decreased, while LH and SHBG increased (75±26 vs. 62±28 pg/mL, 6.6±6.3 vs. 11.2±10.2 mIU/mL, 48±20 vs. 57±23 nmol/L, respectively; p < 0.001), remaining significant after adjusting for baseline age, body mass index (BMI), comorbidities, education, and lifestyle. Men transitioned from biochemical eugonadism (normal FT, LH; 81 vs. 53%) to: compensated (normal FT, high LH; 8 vs. 18%); and overt testicular dysfunction (low FT, high LH; 4 vs. 20%); and rarely hypothalamic-pituitary dysfunction (low FT, non-elevated LH; 6 vs. 8%). At the final visit, TT, FT, and LH were not associated with BMI, diabetes, or glucose levels and did not predict survival. Men with longevity commonly developed biochemical testicular and rarely hypothalamic-pituitary dysfunction with late-life FT decline, which was not associated with metabolic profile or survival.

